# When enough data are not enough to enact policy: The failure to ban chlorpyrifos

**DOI:** 10.1371/journal.pbio.2003671

**Published:** 2017-12-21

**Authors:** Leonardo Trasande

**Affiliations:** 1 Department of Pediatrics, New York University School of Medicine, New York, New York, United States of America; 2 Department of Environmental Medicine, New York University School of Medicine, New York, New York, United States of America; 3 Department of Population Health, New York University School of Medicine, New York, New York, United States of America; 4 NYU Wagner School of Public Service, New York University, New York, New York, United States of America; 5 NYU College of Global Public Health, New York University, New York, New York, United States of America; National Institute of Environmental Health Sciences, United States of America

## Abstract

Strong evidence now supports the notion that organophosphate pesticides damage the fetal brain and produce cognitive and behavioral dysfunction through multiple mechanisms, including thyroid disruption. A regulatory ban was proposed, but actions to end the use of one such pesticide, chlorpyrifos, in agriculture were recently stopped by the Environmental Protection Agency under false scientific pretenses. This manuscript describes the costs and consequences of this policy failure and notes how this case study is emblematic of a broader dismissal of scientific evidence and attacks on scientific norms. Scientists have a responsibility to rebut and decry these serious challenges to human health and scientific integrity.

This Perspective is part of the *Challenges in Environmental Health: Closing the Gap between Evidence and Regulations Collection*.

Organophosphates are acetylcholinesterase inhibitors that were first developed as human nerve gas agents during World War II but were subsequently adapted as insecticides, as they were effective at killing insects via the same mechanism but at much lower exposure concentrations. Rapid urbanization and population growth accelerated the use of these chemicals in homes and agriculture. Though use is increasingly restricted in developed countries to prevent infestations in homes, chlorpyrifos, diazinon, and malathion remain widely used to protect crops.

Not only are alternative methods available to reduce pesticide use (or at least use less toxic alternatives) [1], but evidence has rapidly accelerated documenting adverse effects of organophosphate exposure in general, and chlorpyrifos in particular, especially of low-level exposure in pregnancy and effects on the fetal brain [2,3]. Multiple longitudinal studies have documented consistent decrements in cognitive function in relationship to prenatal exposure, controlling for multiple other potential predictors, such as socioeconomic status and other environmental exposures, such as lead [4,5]. Prenatal exposure has been associated with magnetic resonance imaging findings in children, including frontal and parietal cortical thinning that are consistent with the neurobehavioral deficits identified in psychological testing [6].

Findings in humans are supported by laboratory studies that document not only inhibition of acetylcholinesterase but disruption of thyroid hormone by chlorpyrifos in laboratory studies, especially at low levels of exposure [7–10]. Thyroid hormone has long been known to be crucial for brain development, so much so that thyroid-stimulating hormone analyses are routinely performed on newborns [11]. During pregnancy, subtle changes in free thyroxine within the normal range that would not prompt clinically significant increases in thyroid-stimulating hormone can induce reductions in intelligence quotient (IQ), changes in brain morphology, and even clinically apparent autism and attention deficit hyperactivity disorder [12–18].

It should be noted that health risks of chlorpyrifos have been raised for decades, resulting in a ban on household use of chlorpyrifos in 2000. The ban was timely for scientific reasons, in that it nested a natural experiment within an ongoing birth cohort study at Columbia University in which the research team was studying effects of pesticide exposure on the developing brain. Before the ban, they found decreases in birth weight and length in relationship to levels of chlorpyrifos in newborn cord blood. After the ban, as levels substantially decreased, associations with these strong predictors of adult neurocognitive and cardiovascular outcomes disappeared [19]. Given the ethical concerns that exist with conducting randomized control trials of synthetic chemical exposures, such natural experiments are rare in environmental health research [20]. Data from this Columbia study provide compelling counterargument to those who have noted the potential for alternative explanations of the other observational studies that have been conducted to date. Emerging evidence also suggests prenatal chlorpyrifos exposure may induce tremor detectible in middle childhood [21].

An expert panel recently evaluated the epidemiological and toxicological evidence for effects of this class of chemicals on cognitive deficits and intellectual disability, using rigorous criteria elaborated by the World Health Organization (WHO) and Danish Environmental Protection Agency. They evaluated the toxicological evidence to be strong and the epidemiological evidence to be of moderate-to-high quality. The expert panel rated probability of a causal relationship to be quite high, between 70%–100% [22].

Yet recently, United States Environmental Protection Agency (USEPA) Administrator Scott Pruitt denied a petition to revoke all food residue tolerances for chlorpyrifos, calling it “crucial to U.S. agriculture” and to “ensur[ing] an abundant and affordable food supply for this nation and for the world” [23]. USEPA had proposed to ban all uses of chlorpyrifos in 2015 [24] and reiterated the need for the ban based upon unacceptably high levels of chlorpyrifos identified in food and drinking water and risks posed to women, children, agricultural communities, and workers [25]. The press release suggested that “predetermined results” had been improperly used to guide policy. It cites methodological concerns voiced by the federal advisory committee tasked with reviewing regulatory decisions on insecticides under the Federal Insecticide, Fungicide and Rodenticide Act, but revisions by USEPA staff had already been made to resolve these concerns [26]. A less inflammatory Federal Register statement suggests that chlorpyrifos is the only cost-effective choice for some crops [27].

The emphasis of Administrator Pruitt on the need for sustaining the food supply by using chlorpyrifos in agriculture [23] bears some further comment. While concerns have been raised about the need for pesticides to proverbially “feed the world,” the evidence for superiority of crop yield is not as ironclad as some suggest. Context clearly matters, as a recent meta-analysis of performance comparisons between conventional and organic agriculture suggests, with equivalent yields under good management practices, particular crop types, and growing conditions [28,29]. Let us for a moment assume that there are no alternatives to chlorpyrifos and that it is needed to sustain the global food supply. At the very least, there are serious tradeoffs to consider in such a decision: is keeping children well fed worth their being less smart and able to contribute to the future of the global economy? Administrator Pruitt’s statement on chlorpyrifos is mum on this point.

Researchers at four institutions who led the birth cohort studies did not conspire to select populations or otherwise design their studies with the intention of finding adverse effects of organophosphate exposures on child neurodevelopment [30]. Results were rigorously vetted in peer review by researchers independent of the primary study authors, after successfully having their grant applications reviewed stringently by the National Institutes of Health (NIH) through its own separate peer review process. “Predetermined” that surely is not; the use of “predetermined” should alarm all in the scientific community. In addition, Administrator Pruitt’s decision fails to consider the reality that the cohort of US children born in 2010 lost 1.8 million IQ points and 7,500 children had their IQs shifted into the intellectual disability range as a result of prenatal organophosphate exposures [31].

The costs of Administrator Pruitt’s inaction are also substantial for our economy. While at an individual level an IQ point may not be perceptible except to the keen tools used by the neuropsychologist, a large literature has documented that each IQ point lost translates to a 2% reduction in lifetime economic productivity [32]. On average, children born in the US today are expected to have approximately $1,000,000 in economic productivity, after adding up income over the life course and appropriately discounting for time preference. That equates to roughly $20,000 per IQ point, not to mention the additional educational and healthcare costs (among others) associated with intellectual disability. Together, the 1.8 million IQ point loss in each birth cohort of children will cost the US $44.7 billion annually, assuming future birth cohorts are exposed at current levels [31]. Chlorpyrifos is but one organophosphate pesticide, and few epidemiological studies have measured serum chlorpyrifos, instead measuring the dialkylphosphate metabolites common to the organophosphates. Estimates of disease burden due to organophosphates cannot therefore be parsed to isolate a chlorpyrifos-specific disease burden. Having raised an important caveat, the judgment that chlorpyrifos is the only cost-effective choice for some crops [27] does not consider a very large societal cost associated with lost IQ.

So when are enough data enough to prompt policy action to protect the public? Fifty years ago, during a period of intense debate about the health effects of tobacco, Sir Austin Bradford Hill gave a landmark lecture on criteria for causation. Clearly, exposure must precede effect for us to even consider causality. Hill identified additional criteria that should be considered, such as consistency, exposure–response relations, biological plausibility, effect size, and specificity. What has been easily forgotten from Hill’s lecture is the need for context in considering the totality of evidence. The stakes involved clearly matter; Hill used the example of restricting the use of a drug for morning sickness for pregnant women, suggesting that we might act on “relatively slight evidence” of harm, as “[t]he good lady and the pharmaceutical industry will doubtless survive.” Similarly, for an occupational carcinogen, “fair” evidence would be sufficient. He closes by emphasizing: “All scientific work is incomplete—whether it be observational or experimental…That does not confer upon us a freedom to ignore the knowledge we already have, or to postpone the action that it appears to demand at a given time” [33].

Scientists have a responsibility to speak up when policymakers fail to accept scientific data. They need to emphatically declare the implications of policy failures, even if some of the scientific underpinnings remain uncertain. There is always some uncertainty in estimating exposures from epidemiology studies, especially where there has been exposure to multiple organophosphates, for example. In the case of organophosphates, there is still ample evidence to support a ban given the consistent findings among a large number of epidemiology studies as well as laboratory studies, which suggest a modest or perhaps minimal uncertainty of causation. One could easily replace the example of chlorpyrifos with a host of other synthetic chemicals where policy action remains lagging despite substantial evidence for serious public health concern. These include brominated flame retardants, phthalates, and bisphenols, just to name a few, as documented in recent reports by the Endocrine Society [34] and the WHO and United Nations Environment Programme (UNEP) [35] on endocrine disruptors.

Scientists who raise their voices should be prepared to face criticism from those who have substantial vested interests. A response to the WHO/UNEP report funded by CropLife America (a trade association representing the agricultural pesticides industry), the American Chemistry Council (ACC), as well as Canadian and European trade associations, published critical comments, and these were rebutted [36]—that is all for the good. Individual scientists have been targeted, myself included. A consultant to the ACC and his colleague recently responded to estimates of the disease burden and cost estimates due to organophosphate pesticides and other endocrine disrupting chemicals by labeling the evidence for disease causation as “pseudoscience” [37]. In these attacks on my colleagues and I [38], funding sources are not stated, and no conflicts of interest are declared.

One of the two authors argued that government-funded academic researchers have an incentive to report adverse findings regarding chemical exposures to preserve their careers [39]. The reality is quite different—many peer-reviewed publications use the adage “further research is needed” and are silent about the implications for regulatory action or other opportunities for action. Researchers may actually—and wrongly—be inclined to temper interpretation of study findings because definitive conclusions could preclude grant applications with new studies of chemicals for which an accumulated literature suggests probable causation.

Attacks on scientific norms are likely to continue unabated, be they on climate change, the benefits of vaccines, synthetic chemical exposures, or many other important public health issues. We cannot become numb and tune these attacks out. In an era when fake news is rampant, scientists and journal editors must identify knowledge gaps while documenting the urgency for action. There can of course be debate about the specific action and its urgency. Taking chlorpyrifos off the market will preserve our children’s intellectual potential. The chemical and agricultural industries will survive as they have survived the loss of many chemicals.

## Abbreviations

ACC: American Chemistry Council
IQ: intelligence quotient
NIH: National Institutes of Health
UNEP: United Nations Environment Programme
USEPA: United States Environmental Protection Agency
WHO: World Health Organization
